# Differentiation of invasive ductal and lobular carcinoma of the breast using MRI radiomic features: a pilot study

**DOI:** 10.12688/f1000research.146052.1

**Published:** 2024-02-01

**Authors:** Sudeepta Maiti, Shailesh Nayak, Karthikeya D Hebbar, Saikiran Pendem

**Affiliations:** 1 Department of Medical Imaging Technology, Manipal College of Health Professions, Manipal Academy of Higher Education, Manipal, Karnataka, 576104, India; 2 Department of Radio diagnosis and Imaging, Kasturba Medical College, Manipal Academy of Higher Education, Manipal, Karnataka, 576140, India

**Keywords:** Invasive carcinoma, Radiomic features, MRI Sequences, Magnetic Resonance Imaging (MRI), Noninvasive diagnosis

## Abstract

**Background:**

Breast cancer (BC) is one of the main causes of cancer-related mortality among women. For clinical management to help patients survive longer and spend less time on treatment, early and precise cancer identification and differentiation of breast lesions are crucial. To investigate the accuracy of radiomic features (RF) extracted from dynamic contrast-enhanced Magnetic Resonance Imaging (DCE MRI) for differentiating invasive ductal carcinoma (IDC) from invasive lobular carcinoma (ILC).

**Methods:**

This is a retrospective study. The IDC of 30 and ILC of 28 patients from Dukes breast cancer MRI data set of The Cancer Imaging Archive (TCIA), were included. The RF were extracted from the DCE-MRI sequence using a 3D slicer. The relevance of RF for differentiating IDC from ILC was evaluated using the maximum relevance minimum redundancy (mRMR) and Mann-Whitney test. Receiver Operating Characteristic (ROC) curve analysis was performed to ascertain the accuracy of RF in distinguishing between IDC and ILC.

**Results:**

Ten DCE MRI-based RFs used in our study showed a significant difference (p <0.001) between IDC and ILC. We noticed that DCE RF, such as Gray level run length matrix (GLRLM) gray level variance (sensitivity (SN) 97.21%, specificity (SP) 96.2%, area under curve (AUC) 0.998), Gray level co-occurrence matrix (GLCM) difference average (SN 95.72%, SP 96.34%, AUC 0.983), GLCM interquartile range (SN 95.24%, SP 97.31%, AUC 0.968), had the strongest ability to differentiate IDC and ILC.

**Conclusions:**

MRI-based RF derived from DCE sequences can be used in clinical settings to differentiate malignant lesions of the breast, such as IDC and ILC, without requiring intrusive procedures.

## Introduction

Breast cancer (BC) is the most frequently diagnosed cancer among women worldwide. With an expected 2.3 million new cases, or 11.7% of all cancer cases worldwide in 2020, lung cancer has surpassed lung cancer as the most common cause of cancer incidence. ^1^ According to epidemiological studies, by 2030, there will likely be roughly 2 million BC patients worldwide, according to epidemiological studies. ^2^ Early and precise identification and characterization of cancers are crucial because of their incidence and therapeutic significance.

Mammography and Ultrasonography are frequently used for breast lesion detection, screening and diagnostic purposes. ^3^^,^^4^ Breast Magnetic Resonance Imaging (MRI) is performed regularly to better detect primary and recurrent tumors, characterize them, and assess the patient’s response to therapy. Dynamic contrast-enhanced (DCE) MRI is an important component of the MRI-Breast protocol, which involves serial capture of strong T1 weighted images during intravenous administration of contrast. It provides information about tumor vascularity, and enhancement curves are frequently used to increase specificity (greater than 90%) for diagnosing cancer. ^5^^–^^8^

Radiomics is a popular area of study in the processing and analysis of medical images. Radiomics provides more information than the visual and qualitative patterns that radiologists can see with their unaided eyes by extracting a large number of quantitative imaging features from medical images. On routine imaging examinations performed in cancer patients, radiomic feature (RF) characteristics can non-invasively evaluate intratumoral variability. ^9^^,^^10^ The use of RF in patients with BC is a novel and developing area of translational research. RF in BC has been extensively employed in research settings with the hope that it may eventually improve diagnosis and characterization. ^11^^,^^12^

There are significant methodological variations in research involving RF and artificial intelligence (AI) techniques, such as machine learning (ML) based on radiomics, and there is potential for methodological advancement and standardization to improve study quality in BC. There is a lack of reproducibility and validation in radiomic studies. ^13^ Our literature review revealed a few studies that used RF from the DCE sequence of breast MRI for differentiating malignant lesions such as invasive ductal carcinoma (IDC) and invasive lobular carcinoma (ILC). Hence, our study investigated whether RF obtained from DCE-MRI would aid in the differentiation of IDC and ILC.

## Methods

First, this was a retrospective study. The study was commenced upon approval from the Institutional Ethical Committee of Kasturba Medical College and Hospital, Manipal, India on 6 ^th^ August 2022 (IEC 202/2022). Informed consent was waived since the data was collected from a publicly available database.

### Eligibility criteria

Patients with confirmed histopathology diagnosis of IDC and ILC were included. The cases with artifacts on MRI images were excluded.

### MRI scanning

Patients with confirmed histopathological reports of IDC (30) and ILC (28) from the Duke breast cancer MRI data set of the cancer imaging archive (TCIA) were included in this study ^14^^,^^15^ (underlying data). ^16^ The MRI breast dataset (IDC and ILC) used for this study is publicly available online at https://wiki.cancerimagingarchive.net/; https://doi.org/10.7937/TCIA.e3sv-re93). The Duke breast cancer dataset is composed of a retrospectively collected cohort of 922 biopsy-confirmed invasive breast cancer patients from a single institution (Duke Hospital, Durham, North Carolina, USA) with preoperative MRI from January 1, 2000 to March 23, 2014. The images were acquired using 1.5 Tesla GE (Signa Excite, Signa HDxt) and Siemens (Avanto) MRI scanners. The mean age (years) of the patients with IDC and ILC was 48 ± 11.15 and 59 ± 11 years, respectively. Demographic characteristics are shown in Table 1. Axial T1 dynamic post-contrast (DCE) sequence (gadolinium-based contrast of 15–20 ml) was performed using the image acquisition parameters listed in Table 2.

**Table 1.** Demographic and clinical characteristics of subjects (n = 58).

	Malignant breast lesion categories	
	IDC	ILC
**Subject (n)**	30	28
**Age in years (Mean± SD)**	48 ± 11.15	59 ± 11
**Gender**	Female	

**Table 2.** MRI acquisition parameters of axial T1 dynamic contrast enhancement sequence.

Acquisition parameter	DCE MRI
**Type**	3D
**Sequence**	Gradient
**TR (ms)**	4–6
**TE (ms)**	1–2.5
**Matrix size**	320 × 320
**Slice thickness (mm)**	1.2
**Flip angle (degree)**	10
**Slice spacing (mm)**	5.5

### Image segmentation and RF extraction

**Figure 1.**
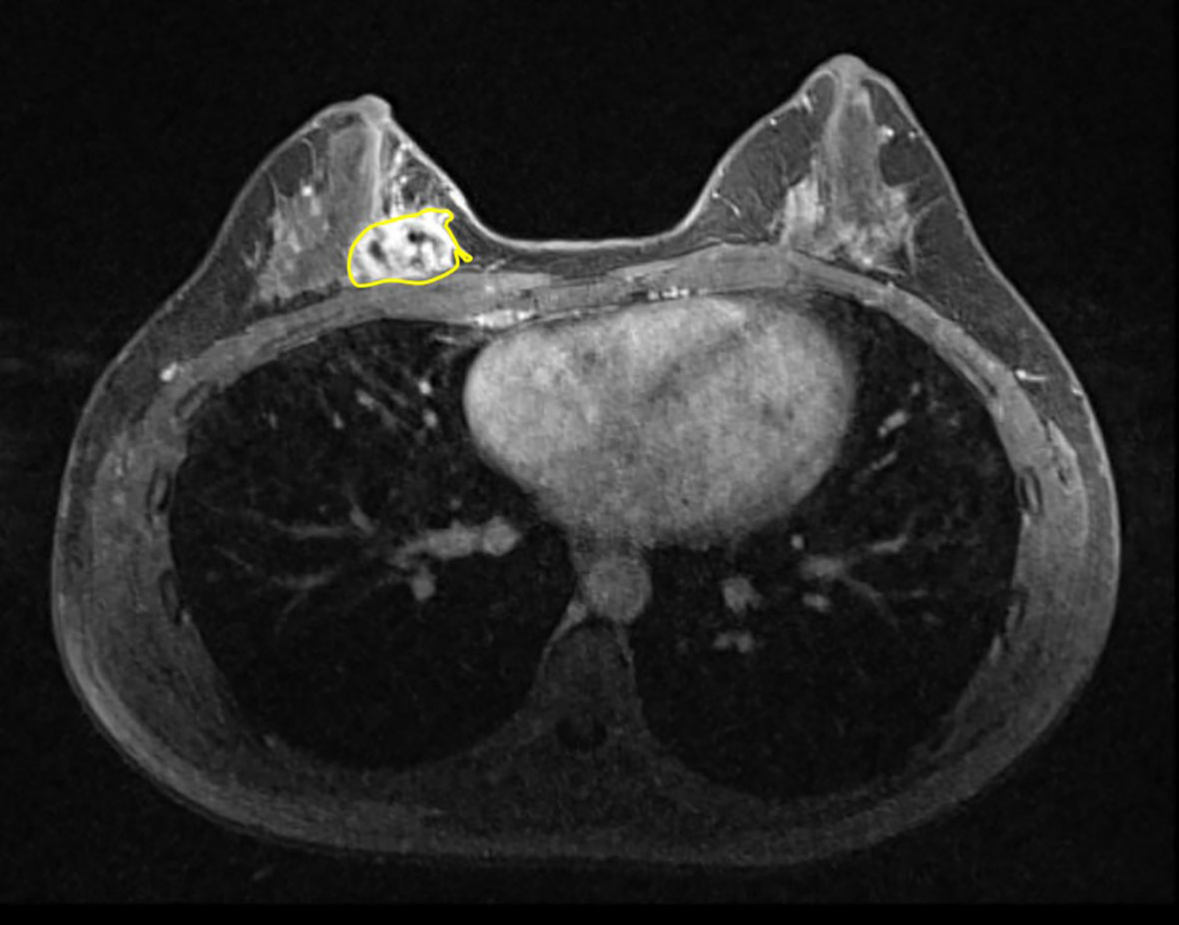
Example of the segmentation of the lesion in axial T1 (3D) dynamic contrast enhanced sequence.

The DICOM MRI images of the Axial T1 DCE were uploaded into 3D slicer (version 4.10.2) and the regions of tumor were manually (slice by-slice) delineated by radiologist who had experience more than 10 years ( Figure 1). The RF was extracted from the axial T1 dynamic contrast-enhanced sequence. ROIs were defined across the entire tumor using DCE-MRI images with the strongest enhancement phase. The observer specifically chose phase (3.92 ± 1.22-on average), to segment the analyzed lesions, out of the six or seven phases offered by the Axial T1 Dynamic contrast enhanced sequences, across different patients, where the breast mass was more visible than in the backdrop. The radiologist was blinded to histopathological reports.

### Feature selection

To determine which RF was the most pertinent and least redundant, the maximum relevance and minimum redundancy (mRMR) approach was used. ^17^ Fifteen RF features were selected for the subsequent analysis ( Table 3).

**Table 3.** The RF selected using mRMR.

S.No.	MRI radiomic features
1	GLDM Gray Level Variance
2	GLDM Gray level Non Uniformity
3	GLDM Low gray level emphasis
4	GLCM Sum Squares
5	GLCM Difference Average
6	GLCM Cluster Tendency
7	GLCM Interquartile Range
8	First Order RMAD
9	First Order Entropy
10	First Order Variance
11	GLRLM Gray Level Variance
12	GLRLM Gray Level Non Uniformity Normalized
13	GLRLM Run Entropy
14	GLSZM Size Zone NonUniformity
15	GLSZM Zone Percentage

### Statistical analysis

Statistical analyses were performed using Statistical Package for Social Sciences (SPSS-20.0) software. The maximum relevance and minimum redundancy (mRMR) approach was applied using Google Colab. The Mann-Whitney U-test was performed to identify significant features for differentiating IDC and ILC on Axial T1 dynamic contrast. Receiver Operating Curve (ROC) analysis was performed to determine the accuracy of RF in differentiating between IDC and ILC. Statistical significance was set at p < 0.001.

## Results

In our study, we analyzed the RF extracted from DCE-MRI. Our study extracted 107 features from the DCE-MRI sequence for each subject. The mRMR technique identified 15 RF features that were relevant for differentiating between IDC and ILC Table 3. Of the 15 selected features, 10 DCE MRI-based RF were significant for differentiating between IDC and ILC. A total of 58 cases were included in the study. The mean age (years) of the patients with IDC and ILC was 48 ± 11.15 and 59 ± 11 years, respectively.

### MRI radiomic features

**Figure 2.**
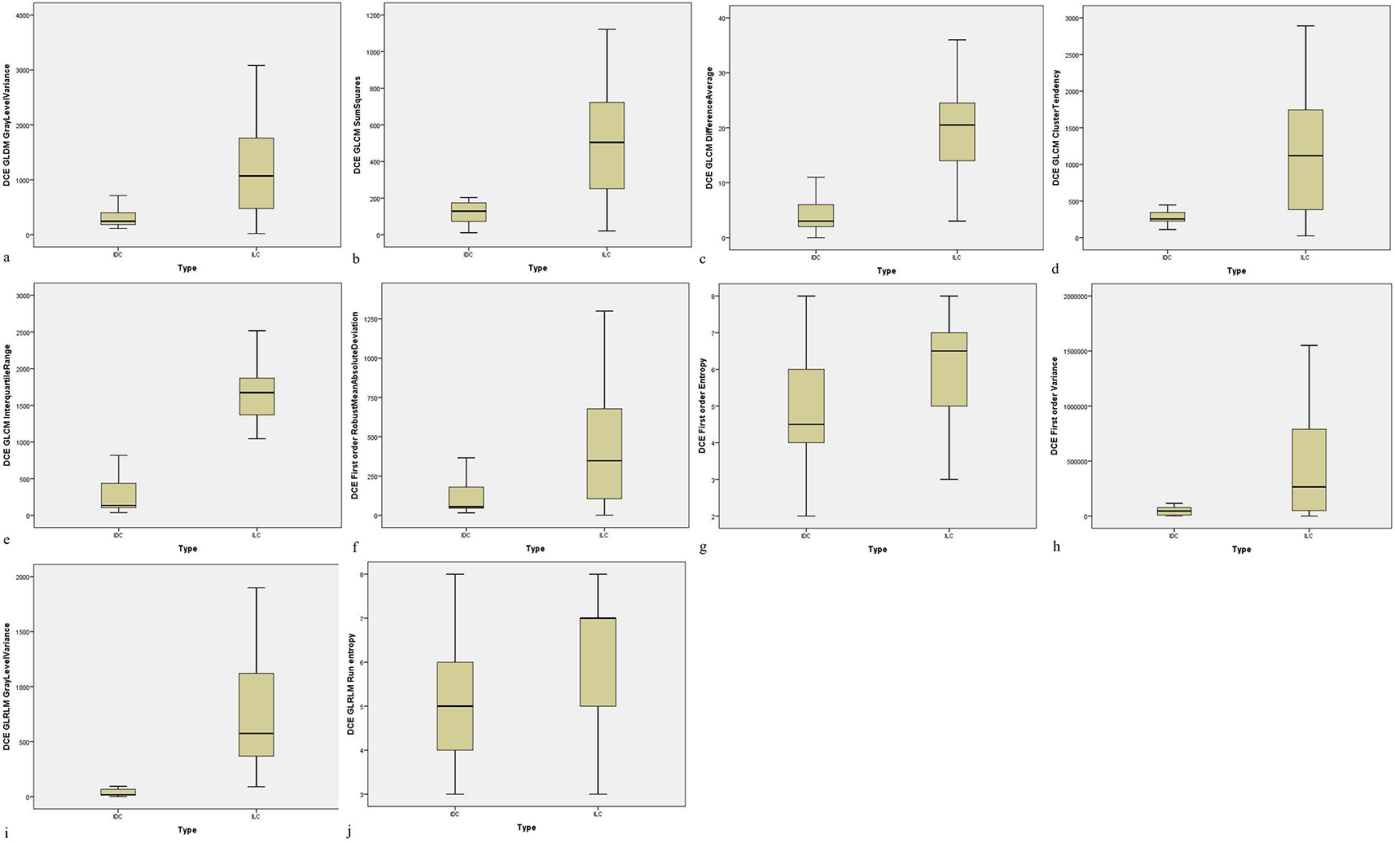
MRI-based significant radiomic features for differentiating IDC and ILC from axial T1 (3D) dynamic contrast enhanced sequence. (a) GLDM Gray Level Variance; (b) GLCM Sum Squares; (c) GLCM Difference Average; (d) GLCM Cluster Tendency; (e) GLCM Interquartile Range; (f) First Order RMAD; (g) First Order Entropy; (h) First Order Variance; (i) GLRLM Gray Level Variance; (j) GLRLM Run Entropy.

For 3D DCE MRI sequence, Gray level dependence matrix (GLDM) gray level variance for IDC and ILC was 288.6 ± 136.4 and 1187.0 ± 342.5 (p < 0.001), Gray level co-occurrence matrix (GLCM) square for IDC and ILC was 118.5 ± 63.15 and 492.4 ± 277.6 (p < 0.001), GLCM difference average for IDC and ILC was 4.472 ± 2.694 and 20.45 ± 10.11 (p < 0.001), GLCM cluster tendency for IDC and ILC was 270.3 ± 81.47 and 1170.8 ± 145.5 (p < 0.001), GLCM interquartile range for IDC and ILC was 286.2 ± 52.8 and 1630.8 ± 332.9 (p < 0.001), first order robust-mean absolute deviation for IDC and ILC was 117.9 ± 18.2 and 403.5 ± 179.0 (p < 0.001), first order entropy for IDC and ILC was 4.776 ± 1.656 and 6.063 ± 1.697 (p < 0.001), first order variance for IDC and ILC was 44324.8 ± 3799.0 and 438604.1 ± 39979.8 (p < 0.001), Gray level run length matrix (GLRLM) gray level variance for IDC and ILC was 35.64 ± 31.20 and 741.40 ± 173.3 (p < 0.001) and GLRLM run entropy for IDC and ILC was 5.496 ± 1.677 and 6.814 ± 1.65 (p < 0.001) ( Table 4 and Figure 2).

**Table 4.** DCE-MRI based radiomic features for differentiating the malignant lesions of the breast.

MRI radiomic features	IDC (n = 30)	ILC (n = 28)	p-value
	(Mean ± SD)		
DCE GLDM Gray Level Variance	288.6 ± 136.4	1187.0 ± 742.5	<0.001
DCE GLCM Sum Squares	118.5 ± 63.15	492.4 ± 277.6	<0.001
DCE GLCM Difference Average	4.472 ± 2.694	20.45 ± 10.11	<0.001
DCE GLCM Cluster Tendency	270.3 ± 81.47	1170.8 ± 745.5	<0.001
DCE GLCM Interquartile Range	286.2 ± 152.8	1630.8 ± 732.9	<0.001
DCE First Order RMAD	117.9 ± 18.2	403.5 ± 279.0	<0.001
DCE First Order Entropy	4.776 ± 1.656	6.063 ± 1.697	<0.001
DCE First Order Variance	44324.8 ± 3799.0	438604.1 ± 39979.8	<0.001
DCE GLRLM Gray Level Variance	35.64 ± 31.20	741.40 ± 473.3	<0.001
DCE GLRLM Run Entropy	5.496 ± 1.677	6.814 ± 1.65	<0.001

### Accuracy measures of radiomic features

**Figure 3.**
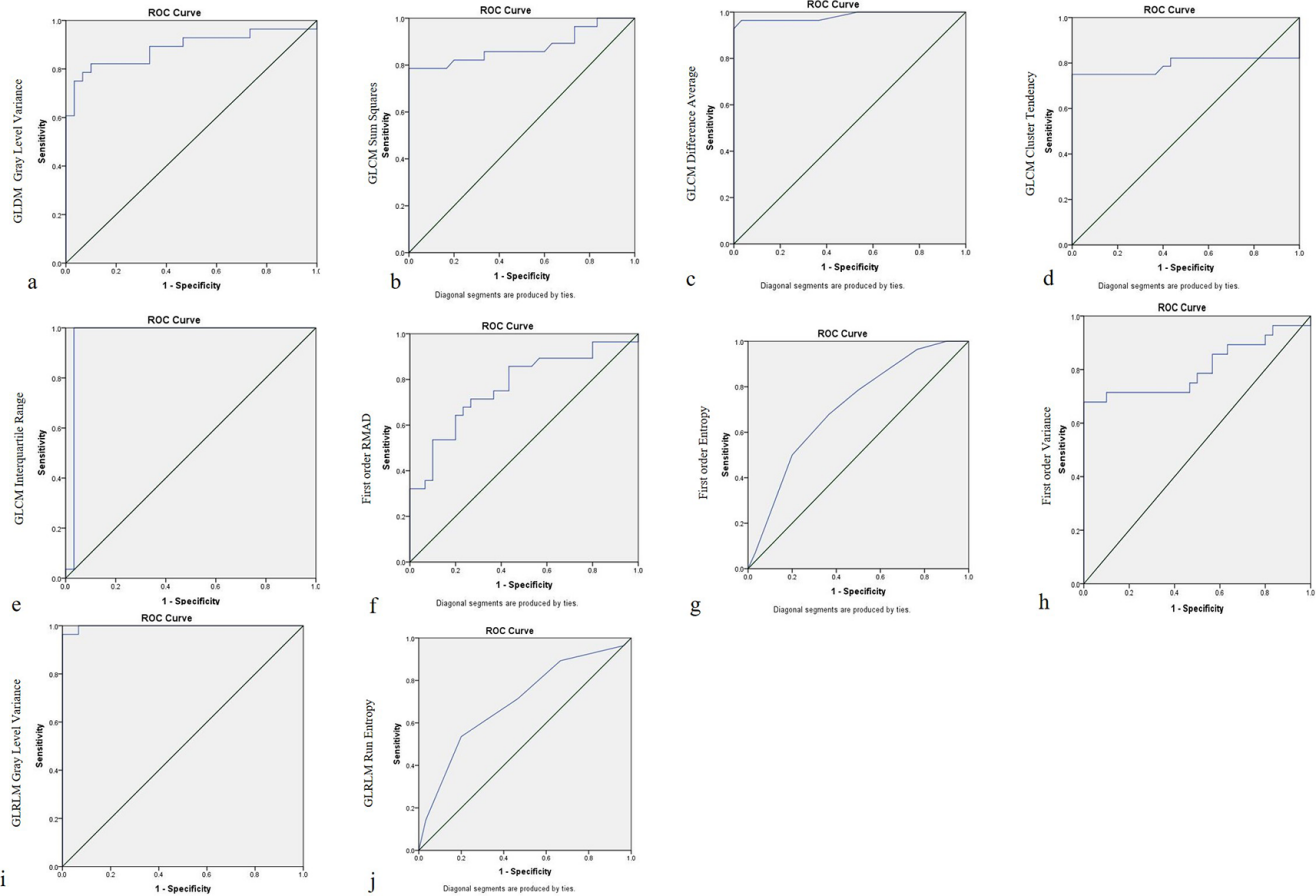
Receiver Operating Characteristic (ROC) curves for differentiating IDC and ILC from Axial T1 (3D) Dynamic contrast enhanced sequence. (a) GLDM Gray Level Variance; (b) GLCM Sum Squares; (c) GLCM Difference Average; (d) GLCM Cluster Tendency; (e) GLCM Interquartile Range; (f) First Order RMAD; (g) First Order Entropy; (h) First Order Variance; (i) GLRLM Gray Level Variance; (j) GLRLM Run Entropy.

The accuracy measures of RF for differentiating between IDC and ILC are listed in Table 5. For 3D DCE MRI sequence, GLRLM gray level variance at cut off value of 42, had higher sensitivity (SN 97.21%), specificity (SP 96.2%), and area under curve (AUC 0.998; GLCM interquartile range at cut off value of 357 had higher SN (95.24%), SP (97.31%), and AUC of 0.968; and GLCM difference average at a cut off value of 6.5, had higher SN 95.72%, SP 96.34% and AUC of 0.983 compared to GLDM gray level variance (SN 91.02%, SP 89.72%), GLCM sum squares (SN 85.91%, SP 82.72%), first order variance (SN 81.54%, SP 82.72%), GLCM cluster tendency (SN 81.77%, SP 80.13%), first order RMAD (SN 80.12%, SP 79.23%), first order entropy (SN 75.24%, SP 72.23%), GLRLM run entropy (SN 75.47%, SP 77.83%) ( Figure 3).

**Table 5.** Area under curve, sensitivity, specificity, and cut-off value for MRI based Radiomic features for differentiating malignant lesions of breast (IDC and ILC).

	Radiomic Features	Area under the curve	Sensitivity (%)	Specificity (%)	Cut off value
**DCE MRI**	DCE GLDM Gray Level Variance	0.887	91.02	89.72	408.5
	DCE GLCM Sum Squares	0.877	85.91	82.72	169.5
	DCE GLCM Difference Average	0.983	95.72	96.34	6.5
	DCE GLCM Cluster Tendency	0.792	81.77	80.13	334
	DCE GLCM Interquartile Range	0.968	95.24	97.31	357
	DCE First Order RMAD	0.768	80.12	79.23	216
	DCE First Order Entropy	0.709	75.24	72.23	5.5
	DCE First Order Variance	0.805	81.54	82.72	780637
	DCE GLRLM Gray Level Variance	0.998	97.21	96.2	42
	DCE GLRLM Run Entropy	0.698	75.47	77.83	5.508

## Discussion

In the current study, we explored the usefulness of DCE-based RF compared to MRI for differentiating malignant lesions of the breast, such as IDC and ILC. Breast MRI is superior to mammography and ultrasonography for early detection of BC. IDC and ILC are the most common subtypes of malignant cancer. Differentiation of ILC from IDC is quite difficult, as there are very few differences between them. The morphological and dynamic contrast kinetic characteristics of IDC and ILC did not differ considerably from each other. The correct identification of ductal and lobular carcinoma helps in the improved management and overall survival analysis of the patient. ^18^^,^^19^

Previous studies have focused on differentiating benign and malignant lesions of the breast using radiomic models in MRI. ^20^^–^^22^ However, few studies have been conducted to differentiate invasive carcinomas of the breast, such as IDC and ILC, using RF from dynamic contrast sequences. The integration of radiomic models in normal radiological practice will be extremely beneficial for non-invasive diagnosis and clinical management of invasive BC in the future.

Our study found ten RF useful in differentiating IDC and ILC. We noticed that categories such as GLDM, GLCM, first order, and GLRLM showed significant differences in differentiating ductal and lobular carcinoma of the breast. Of these categories, GLRLM (Gray level variance AUC 0.998) and GLCM difference average) and interquartile range features were the best predictors for differentiation between IDC and ILC. GLCM features consider how pixels are arranged in space. These features describe the texture of a picture by calculating the frequency of pixel pairings with distinct values and a specific spatial relationship. GLRLM features are used to describe the length of successive pixels with the same grey level value in terms of pixels. GLCM and GLRLM are important markers for assessing tumor heterogeneity and for better characterization of malignant subtypes.

Waugh *et al*. ^23^ in their study noticed that all co-occurrence RF had higher accuracy (71.4% and AUC –0.745) in differentiating ductal and lobular carcinoma than entropy features (64.7% and AUROC –0.632). Holli *et al*. ^24^ noticed that the co-occurrence RF of subtraction first images was more statistically significant than that of other features ( Table 6). They also achieved a classification accuracy of 100% using first subtraction and contrast series using nonlinear and linear discriminant analyses. Our study also noticed that co-occurrence matrix features, such as sum squares, difference average, and cluster tendency, exhibited good accuracy in differentiating ductal and lobular carcinomas. In addition to co-occurrence matrix features, we also noticed additional categories showing statistically significant differences, which were not reported by Holli *et al*. ^24^ and Waugh *et al*. ^23^

**Table 6.** Comparison of radiomic features based breast lesion classification among various studies.

Author (Year)	Our study	Fusco *et al*. ^20^ (2022)	Niu *et al*. ^21^ (2022)	Lafci *et al*. ^25^ (2022)	Militello *et al*. ^22^ (2021)	Waugh *et al*. ^23^ (2016)	Holli *et al*. ^24^ (2010)
Radiomic features (RF) obtained	107	48	105	43	107	220	300
Lesions	IDC and ILC	Benign & Malignant	Benign & Malignant	IDC (Luminal A and B)	Benign and Malignant	IDC and ILC	IDC and ILC
RF Category	Shape, GLDM, GLCM, First order, GLRLM, GLSZM, NGTDM	First and Second order features	Shape, GLDM, GLCM, First order, GLRLM, GLSZM, NGTDM	Conventional, Shape, Histogram, GLCM, GLRLM, NGLDM, GLZLM	Shape, Firstorder, GLCM, GLRLM, GLSZM, GLDM, NGTDM	Co-occurrence matrix	Co-occurrence matrix
Modality	MRI	MRI and X-ray	Digital Mammogarphy, Digital breast Tomosynthesis, MRI	MRI	MRI	MRI	MRI
Significant Radiomic features	GLDM Gray Level Variance, GLCM Sum Squares, GLCM Difference Average, GLCM Cluster Tendency, GLCM Interquartile Range, First Order RMAD, First Order Entropy, First Order Variance, GLRLM Gray Level Variance, GLRLM Run Entropy, Shape voxel volume and mess voulme	IQR, Variance, Correlation, Kurotsis, Skewness, Range, Energy, Entropy, GLN – GLRLM, GLN-GLSZM	GLSZM ZonePercentage, Firstorder Skewness, GLRLM ShortRunLowGrayLevelEmphasis, GLCM Imcl, GLCM ClusterShade, GLCM InverseVariance, Glcm MCC	Histogram: *skewness,* Shape: *volume-ml, volume-voxel,* GLCM: *entropy.log10, entropy.log2, energy* GLRLM: *GLNU, RLNU, HGRE,* NGLDM: *busyness,* GLZLM: *GLNU, HGZE, ZLNU, SZE*	GLCM Joint average, GLRLM Short run emphasis, Shape 3D Least axis length, Shape 3D Flatness, GLRLM Long run low gray level emphasis, GLCM Joint energy, Shape 3D Elongation, GLSZM Size zone non uniformity	Entropy features	GLCM based entropy features

A study by Fusco *et al*. ^20^ observed that kurtosis and skewness (AUC = 0.71) in X-ray mammography and range, energy, entropy, and gray-level non-uniformity (GLN) of the GLRLM from DCE-MRI were the best predictors for differentiating benign and malignant lesions. Niu *et al*. ^21^ they studied the accuracy of RF extracted from digital mammography (DM), digital breast tomosynthesis (DBT), diffusion (DWI), and DCE MRI for the characterization of breast lesions and noticed that the RF extracted from DWI and DCE MRI yielded higher AUC, SN, and SP with DCE having the upper hand compared to DWI, and lower AUC, SN, and SP were noted from DM. Militello *et al*. ^22^ reported that shape-based features such as least axis length, flatness, and elongation, GLCM-based features such as joint energy, GLRLM features such as short run emphasis, and Gray level size zone (GLSZM) of size-non-uniformity from DCE-MRI exhibited the highest SN and SP in characterizing breast lesions.

A study by Lafci *et al*. ^25^ noticed that Gray level zone length matrix (GLZLM) features had the highest accuracy (AUC = 0.718) in distinguishing Luminal A and B types of ductal carcinoma. They also noted that Luminal B tumors had a larger volume than luminal A tumors as they are aggressive and require intense chemotherapy, and observed shape-based features such as voxel volume showed significant differences between A and B. In our study, we also noted a larger voxel volume for ILC (5144 ± 306.5) than for IDC (3899 ± 684.3); however, we did not notice a statistically significant difference because of the smaller sample size. Literature suggests that ILC has a larger volume and is more aggressive compared to IDC. ^26^

Our study has the following limitations. First, we did not utilize machine learning and deep learning classifiers for the prediction of ductal and lobular carcinomas due to the small sample size. Second, longitudinal studies with large sample sizes and machine learning methods were used to further validate the results. Third, as the RF is obtained using manual segmentation, it is time-consuming and subjective.

## Conclusions

Classification of BC into histological subgroups is a dynamic process. Our study suggested an RF-based method for ductal and lobular carcinoma characterization using T1 dynamic contrast sequence, which might give radiologists additional value for decision making in a noninvasive method and could be utilized clinically for malignant BC classification.

## Data Availability

Duke breast cancer MRI data set used for this study are publicly available in the cancer imaging archive (14,15) at https://doi.org/10.7937/TCIA.e3sv-re93 under the terms of the http://creativecommons.org/licenses/by/3.0/ or the https://creativecommons.org/licenses/by/4.0/. There were no changes made to the dataset. Figshare: Underlying data for ‘Differentiation of invasive ductal and lobular carcinoma of the breast using MRI radiomic features: a pilot study’, ‘RF for IDC and ILC-F1000’, https://doi.org/10.6084/m9.figshare.24792693. ^16^ This project contains the following underlying data:
- Demographic characteristics and RF of IDC and ILC (Spread Sheet) - MRI images (DICOM) Data are available under the terms of the Creative Commons Attribution 4.0 International license (CC-BY 4.0).
