# Supplementary material for: Differentiation of invasive ductal and lobular carcinoma of the breast using MRI radiomic features: a pilot study
Source: F1000Res. 2024 Mar 14;13:91. Originally published 2024 Feb 1. [Version 2] doi: 10.12688/f1000research.146052.2 (PMC10988200; doi:10.12688/f1000research.146052.2)
Supplement: Peer review history

# Reviewer report: Reviewer response for version 2

**DOI:** 10.5256/f1000research.163618.r255977

**Published:** 2024-04-02

**Authors:** Mubarak Taiwo Mustapha

**Affiliations:** 1 Near East University, TRNC Mersin, Nicosia, Turkey

All previous comments have been addressed. I have no further comments.

Is the work clearly and accurately presented and does it cite the current literature?

Yes

If applicable, is the statistical analysis and its interpretation appropriate?

Yes

Are all the source data underlying the results available to ensure full reproducibility?

Yes

Is the study design appropriate and is the work technically sound?

Yes

Are the conclusions drawn adequately supported by the results?

Yes

Are sufficient details of methods and analysis provided to allow replication by others?

Yes

Reviewer Expertise:

Artificial Intelligence.

I confirm that I have read this submission and believe that I have an appropriate level of expertise to confirm that it is of an acceptable scientific standard.

# Reviewer report: Reviewer response for version 2

**DOI:** 10.5256/f1000research.163618.r255978

**Published:** 2024-03-28

**Authors:** Avantsa Rohini

**Affiliations:** 1 Radiodiagnosis and Imaging, MNR Medical college and Hospital, Sangareddy, Telangana, India

The manuscript is Approved. No more changes are required.

Is the work clearly and accurately presented and does it cite the current literature?

Yes

If applicable, is the statistical analysis and its interpretation appropriate?

I cannot comment. A qualified statistician is required.

Are all the source data underlying the results available to ensure full reproducibility?

Yes

Is the study design appropriate and is the work technically sound?

Yes

Are the conclusions drawn adequately supported by the results?

Yes

Are sufficient details of methods and analysis provided to allow replication by others?

Yes

Reviewer Expertise:

RADIO DIAGNOSIS AND IMAGING

I confirm that I have read this submission and believe that I have an appropriate level of expertise to confirm that it is of an acceptable scientific standard.

# Reviewer report: Reviewer response for version 1

**DOI:** 10.5256/f1000research.160089.r243255

**Published:** 2024-02-28

**Authors:** Mubarak Taiwo Mustapha

**Affiliations:** 1 Near East University, TRNC Mersin, Nicosia, Turkey

1. **Title and Abstract**:
  - The title effectively summarizes the main objective of the study.
  - The abstract provides a clear overview of the study's background, methods, results, and conclusions. It effectively communicates the main findings. However, it lacks specific details on the radiomic features analyzed and the statistical methods used.
2. **Introduction**:
  - The introduction provides relevant background information on breast cancer and the importance of early and precise identification of breast lesions.
  - It effectively sets the stage for the study and highlights the potential clinical significance of the research. However, it could be improved by providing more context on the challenges in accurately diagnosing invasive ductal carcinoma (IDC) and invasive lobular carcinoma (ILC) and discussing the limitations of current diagnostic methods.
3. **Methods**:
  - The methods section provides a detailed description of the study design, patient population, MRI imaging protocol, and radiomic feature extraction methods.
  - The inclusion and exclusion criteria are clearly stated, and the data source is properly cited. However, the rationale for the sample size and radiomic feature selection criteria should be provided.
  - The use of the mRMR approach and Mann-Whitney U-test for feature selection and statistical analysis is appropriate. However, more details on the statistical analysis methods used for ROC curve analysis would be beneficial.
4. **Results**:
  - The results section presents the findings of the study, demonstrating significant differences in MRI radiomic features between IDC and ILC.
  - The results are clearly presented, but more specific quantitative results, such as effect sizes, confidence intervals, and p-values, would enhance the interpretation of the findings.
5. **Discussion**:
  - The discussion interprets the findings in the context of existing literature and highlights the potential clinical implications of using MRI radiomic features to differentiate between IDC and ILC.
  - The discussion acknowledges the limitations of the study but could be expanded to discuss potential biases, reproducibility issues, and future research directions in more detail.
6. **Conclusion**:
  - The conclusion summarizes the key findings of the study but could be strengthened by emphasizing the clinical significance of the results and suggesting potential implications for breast cancer diagnosis and treatment.
  - It would be beneficial to provide a brief summary of the study's limitations and future research directions in the conclusion section.

Is the work clearly and accurately presented and does it cite the current literature?

Yes

If applicable, is the statistical analysis and its interpretation appropriate?

Yes

Are all the source data underlying the results available to ensure full reproducibility?

Yes

Is the study design appropriate and is the work technically sound?

Yes

Are the conclusions drawn adequately supported by the results?

Yes

Are sufficient details of methods and analysis provided to allow replication by others?

Yes

Reviewer Expertise:

Artificial Intelligence.

I confirm that I have read this submission and believe that I have an appropriate level of expertise to confirm that it is of an acceptable scientific standard, however I have significant reservations, as outlined above.

# Reviewer report: Reviewer response for version 1

**DOI:** 10.5256/f1000research.160089.r243253

**Published:** 2024-02-19

**Authors:** Avantsa Rohini

**Affiliations:** 1 Radiodiagnosis and Imaging, MNR Medical college and Hospital, Sangareddy, Telangana, India

This is a very good article. The concept and idea of differentiating invasive ductal carcinoma and invasive lobular carcinoma prior to invasive test based on MRI radiomic features is interesting, and encouraging. The results of the study might encourage future studies and help in reducing the time gap between diagnosis and treatment of  breast cancer.

Is the work clearly and accurately presented and does it cite the current literature?

Yes

If applicable, is the statistical analysis and its interpretation appropriate?

I cannot comment. A qualified statistician is required.

Are all the source data underlying the results available to ensure full reproducibility?

Yes

Is the study design appropriate and is the work technically sound?

Yes

Are the conclusions drawn adequately supported by the results?

Yes

Are sufficient details of methods and analysis provided to allow replication by others?

Yes

Reviewer Expertise:

RADIO DIAGNOSIS AND IMAGING

I confirm that I have read this submission and believe that I have an appropriate level of expertise to confirm that it is of an acceptable scientific standard.
